# Gene duplication in the genome of parasitic *Giardia lamblia*

**DOI:** 10.1186/1471-2148-10-49

**Published:** 2010-02-17

**Authors:** Jun Sun, Huifeng Jiang, Roberto Flores, Jianfan Wen

**Affiliations:** 1 State Key Laboratory of Genetic Resources and Evolution, Kunming Institute of Zoology, Chinese Academy of Sciences (CAS), Kunming, Yunnan 650223, PR China; 2 Graduate School of Chinese Academy Sciences, Beijing 100039, PR China; 3 Division of Nutritional Sciences, Cornell University, Ithaca, NY 14853, USA

## Abstract

**Background:**

*Giardia *are a group of widespread intestinal protozoan parasites in a number of vertebrates. Much evidence from *G. lamblia *indicated they might be the most primitive extant eukaryotes. When and how such a group of the earliest branching unicellular eukaryotes developed the ability to successfully parasitize the latest branching higher eukaryotes (vertebrates) is an intriguing question. Gene duplication has long been thought to be the most common mechanism in the production of primary resources for the origin of evolutionary novelties. In order to parse the evolutionary trajectory of *Giardia *parasitic lifestyle, here we carried out a genome-wide analysis about gene duplication patterns in *G. lamblia*.

**Results:**

Although genomic comparison showed that in *G. lamblia *the contents of many fundamental biologic pathways are simplified and the whole genome is very compact, in our study 40% of its genes were identified as duplicated genes. Evolutionary distance analyses of these duplicated genes indicated two rounds of large scale duplication events had occurred in *G. lamblia *genome. Functional annotation of them further showed that the majority of recent duplicated genes are VSPs (Variant-specific Surface Proteins), which are essential for the successful parasitic life of *Giardia *in hosts. Based on evolutionary comparison with their hosts, it was found that the rapid expansion of VSPs in *G. lamblia *is consistent with the evolutionary radiation of placental mammals.

**Conclusions:**

Based on the genome-wide analysis of duplicated genes in *G. lamblia*, we found that gene duplication was essential for the origin and evolution of *Giardia *parasitic lifestyle. The recent expansion of VSPs uniquely occurring in *G. lamblia *is consistent with the increment of its hosts. Therefore we proposed a hypothesis that the increment of *Giradia *hosts might be the driving force for the rapid expansion of VSPs.

## Background

*Giardia *are a group of flagellated unicellular protists which are the most common infective parasites of a number of vertebrates. For example, *G. lamblia *is a common human parasite. In the United States, about 20,000 cases of giardisis are reported each year [1]. Aside from being a prevalent pathogen, in the last two decades *G. lamblia *has caught a lot of attentions, as being the most primitive eukaryotes [2]. Phylogenetic and cellular evidence indicate that this organism might branch away from the ancestor of extant eukaryotes around the endosymbiotic origin of mitochondria in eukaryotes [2-5]. Therefore before the emergence of multicellular animals, *G. lamblia *may have survived freely in the world for several hundred million years [6]. It suggested that later on it developed the ability to successfully parasitize vertebrates, as it is now recognized as one of the most prevalent intestinal parasites in a variety of vertebrates from amphibians to mammals [7]. An intriguing question is how this ancient eukaryote became an obligate parasite in the later multicellular animals. The draft genome sequence of *G. lamblia *provides us an opportunity to uncover what genomic features resulted in its parasitic lifestyle [8]. Comprehension of how the parasitic ability developed would not only be of evolutionary biological significance, but also shed light on the mechanism of giardisis.

Genetic novelties emerge in organisms by creation of new genes through three major mechanisms: de novo creation, lateral gene transfer and gene duplication [9,10]. The Origin of new genes de novo in *G. lamblia *is impossible to detect because of the deficiency of close relatives in the lineage. Lateral gene transfer (LGT), which is a predominant force of acquisition of new genes in many microorganisms [11], may play an important role for the adaptive evolution of *G. lamblia *in animal intestines because half of the 15 LGT genes identified are associated with its surveillance in an anaerobic environment [12]. However, gene duplication, which has long been thought to be the primary mechanism in producing resources for the origin of evolutionary novelties, has not yet been thoroughly studied in *G. lamblia*. The most obvious contribution of gene duplication to organisms is that it provides genetic material to generate neo-function or sub-function while maintaining the original function of duplicated genes [9,13]. Moreover, the generation of duplicated genes can increase genetic robustness within cellular networks [14]. The dynamic evolution of duplicated genes inflects adaptive evolution of organisms under varying environments [15,16]. Therefore there is no doubt that gene duplication is extremely pervasive, conducting function in almost all organisms from prokaryotes to eukaryotes [9,13].

Previously, many studies on fungi, plants and animals have shown that gene duplication contributes novelties for their adaptive evolution [17-21]. In order to investigate the impact of gene duplication on the parasitic lifestyle of *G. lamblia*, we surveyed and depicted the evolutionary relationships of all the duplicated genes in its genome. Our results showed that two rounds of large scale duplication events took place in the evolutionary process of *G. lamblia*. Furthermore, most of the recent duplicated genes in the second round duplication events are VSP genes, which are essential for the parasitic properties of *G. lamblia *that utilizes them to evade the host's immune response [22]. Largely expanded VSP genes are helpful to parasitize a variety of hosts, because they allow *G. lamblia *to enact a more complex regulation of VSPs in different hosts and at different times [23-25].

## Results

### Identification of Gene duplication events in *G. lamblia*

Gene duplication and subsequent divergence of the duplicated copies provide opportunities to generate neo-function for adaptive evolution of organisms in varying environments. Identification of duplicated genes is valuable to understand the adaptive evolution of organisms. In order to identify all putative gene duplication events in *G. lamblia*, a very ancient organism which have survived in the world for several billion years [6], a global survey of protein similarities was conducted using the BLASTP program with loose parameters (E-value < 10^-4^) [26]. After all-against-all alignments for the entirety of proteins in *G. lamblia*, we detected 2,403 duplication genes which cover about 40% of the total proteins in *G. lamblia*. Duplicated genes usually can be classified into tandem, segmental and dispersed duplicates. According to the location of duplicated genes in the assembled contigs, we found only 23 tandem duplicated genes whose original gene and duplicated copy are tandemly located on the same contig. Additionally eight genes were involved in segmental duplication events, which resulted in two or more duplicated genes located in the same contig (Additional file 1: The list of 2,403 duplicated genes). These results imply that the majority of duplication events happened dispersedly.

**Figure 1:**
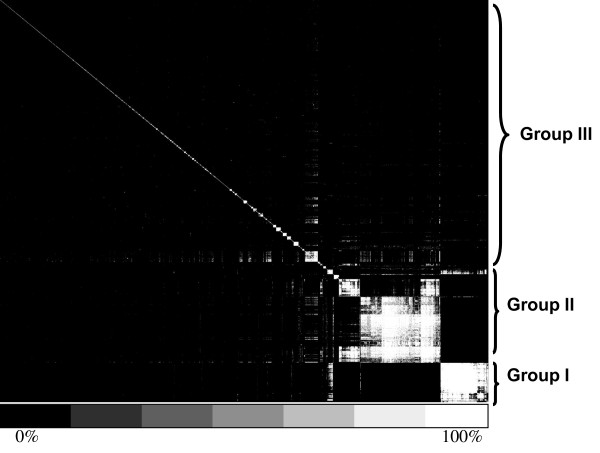
**Cluster analysis of duplicated genes**. The similarities of duplicated genes were denoted with different colors. As the bottom bar showed, the black means the similarity value is 0% and the white means the similarity value is 100%. Group I and Group II were defined based on the cluster results. As a control for comparing analysis, the rest proteins were put together as Group III.

In order to further analyze the evolutionary scenario of these duplication events, subsequently, a cluster analysis was done based on sequence divergence of the duplicated genes. Briefly, we constructed a matrix with 2,403 rows and columns where each represented a duplicated gene. The amino acid similarities for each gene pair in the row and column were then extracted from the BLASTP results. Based on this matrix, a hierarchical clustering was conducted by the program AGNES (agglomerative hierarchical clustering algorithms) where proteins are clustered closer if their protein sequences have higher similarity (Materials and Methods) [27]. Interestingly, as shown in figure 1, roughly 30% of the duplicated genes were classified into two large duplicated groups: 235 genes in Group I and 500 genes in Group II. The rest of the 1,668 genes formed many small gene groups. A control dataset which contains these 1,668 genes (Group III) was also constructed, in order to see if the evolutionary pattern of the two large groups is different from these small groups. Comparison of average similarities among genes in the three groups showed that the average similarity for genes from Group I (35.26 ± 0.09) is significantly higher than those from Group II (31.32 ± 0.02) and III (29.03 ± 0.18). This implies perhaps the majority of duplication events in Group I took place more recently than those in Groups II and III did.

### Two rounds of large scale duplication events happened in *G. lamblia*

**Figure 2:**
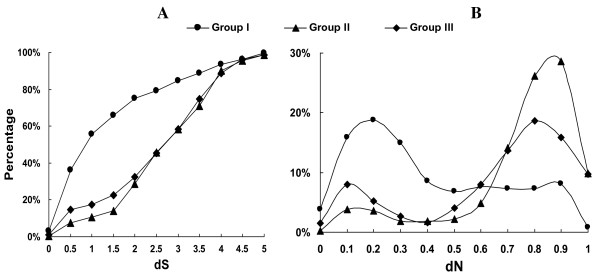
**Distribution of evolutionary distances for duplicated gene**. A) Cumulative distribution of dS for duplicated genes in Group I, II and III. B) dN distribution of duplicated genes in *G. lamblia*. X axis means values for evolutionary distances (dS and dN) and Y axis displayed the percentage of duplicated genes.

In order to further clarify the evolutionary order of the duplication events in each group, we defined the best hit for each duplicated gene as its direct parental gene (Materials and Methods). Due to a large divergence in sequence, it is impossible to detect the parental genes for some ancient duplicated genes. Fortunately, we identified 1,907 pairs of parent-daughter relationships among all duplicated genes in *G. lamblia*, but the rest of the 496 duplicated genes lacked detectable parental genes. To gauge the evolutionary distances for each parent-daughter pair, we used non-synonymous distance (dN) and synonymous distance (dS) [28,29] (See Additional file 1 for the list of dN and dS value for all duplicated pairs). As shown in figure 2A, most genes in Group I were created very recently compared with the genes in Group II and III. For example 56% of the genes in Group I have dS smaller than 1, whereas the genes with such a low dS in Group II and III are only 11% and 17%, respectively. Thus, more than half of the duplicated genes in Group I were originated recently. Since the larger dS values for most duplicated genes in Group II and Group III might result from reverse mutations at synonymous sites, the evolutionary distances for a large number of duplicated genes in Group II and III cann't only be based on dS. As a result, we turned to dN.

Although positive selection would accelerate non-synonymous mutation rate in a short period, in the long term, especially for duplicated genes in Group II and III with such a large divergence, dN is possible to use as an index of evolutionary time. Therefore we used dN to characterize the duplication events within the three groups. Unexpectedly, the distribution of dN values for the totality of duplicated genes clearly shows that there were two rounds of gene duplication events in the genome of *G. lamblia *(Figure 2B): the first round of duplication events that were mainly enriched in Groups II and III happened earlier, while the second round of events focusing on Group I occurred very recently. Larger dN values in Groups II and III maybe the result of functional relaxation of the genes within both groups. However functional relaxation of genes in Groups II and III is not enough to explain why the dN values in both groups are higher than those in Group I, due to no significant difference between their dN/dS ratio (*P*-value of t-test is 0.5 between dN/dS from Group I and II and 0.15 between Group I and III). Based on the distribution of dN values, we arbitrarily defined two types of duplicated genes in each group: recent duplicated gene (RDG) with dN < 0.5 and ancient duplicated gene (ADG) with dN > 0.5. Thus 68.5% of the genes in Group I belongs to RDG. This proportion is much higher than those in Group II (13.4%) and III (16.5%). Therefore no matter what methods we utilize, duplicated genes in Group I seem much younger than those in Groups II and III.

### Recently duplicated genes in *G. lamblia *are significantly biased towards VSP genes

The current adaptation of an organism relies on recent genomic contents. We are interested in testing if the recently duplicated genes in Group I are related to the parasitic life in *G. lamblia*. In order to do this, we first annotated functional domains for duplicated genes in each group using the Pfam database (Materials and methods). For the two large duplicated gene groups, 86% of the duplicated genes in Group I were annotated as VSP function, while in Group II 72% of the genes were annotated as Ank function and 27% as Pkinase (See Additional file 1 for functional domain annotation). As we mentioned above, more recent duplication genes are rich in Group I than those in Group II and III. By counting RDG and ADG in each group, we found that in Group II more than 80% of Ank genes and Pkinase belong to ADG, while 74% VSP genes belong to RDG in Group I.

**Figure 3:**
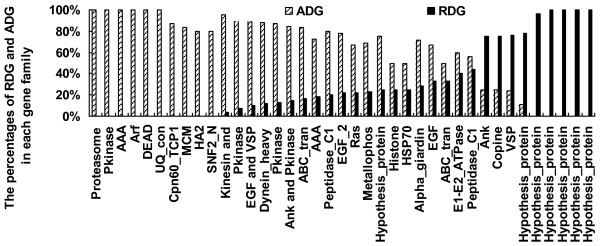
**Rates of RDG and ADG in functional categories**. The proportions of RDG and ADG in each family with more than 4 duplicated genes in *G. lamblia *were displayed. X axis means functional categories and Y axis denotes the proportion.

Secondly, in order to study the functional distribution of duplicated genes in detail we clustered all proteins from *G. lamblia *into different gene families by Tribe-MCL [30]. We checked the proportion of RDG and ADG in each gene family to see if functional bias occurred between RDG and ADG. As shown in figure 3, functional bias between RDG and ADG is obvious in most of the gene families (here only families with more than 4 members were listed, Additional file 2 listed the ratio of RDG and ADG in each gene family). Many gene families with important functions had been duplicated long before such as: the Ank domain which possibly plays roles for localization in cells, the Pkinase which may be functional in signal transduction and protein degradation. Interestingly, two types of motor proteins (Kinesin and Dynein) were also significantly enriched in the ADG. It may be possible that such an enrichment of motor proteins were related to the earlier adaptation of *G. lamblia*, which moved in water by its flagella [31,32]. In addition, six out of the nine gene families enriched in RDG were annotated as hypothesis proteins and the additional three families were Ank, Copine and VSP. Although Ank function is also enriched in RDG, there are only four members in the family, as well as in Copine. It was noticed that six gene families were annotated as hypothesis proteins. Further functional studies of these families would provide more valuable insights into the recently adaptive evolution of *G. liamblia*. Most of the VSPs were created very recently, which is consentient with our results above. Many studies have shown that VSPs play a profound role in antigenic variation of *G. lamblia*, which expresses only one of the VSP genes at a particular time and spontaneously switches to varying VSP genes every 6-13 generations [23]. Therefore numerous duplications of VSPs are indispensable for *G. lamblia *to be able to infect a variety of hosts.

### The rapid expansion of VSPs in *G. lamblia *may be consistent with the evolutionary radiation of placental mammals

Evolutionary arms-race is an important driving force for the adaptive evolution [33-35]. The most important thing for a successful parasite in this race is to develop a mechanism to allow antigenic variation to escape arrest from the host immune system. VSPs are an essential gene family in *G. lamblia *to carry out antigenic variation. Therefore, we asked if the rapid expansion of VSPs in *G. lamblia *is associated with the evolution of its hosts. Comparison of the evolution rate of VSPs in *G. lamblia *with the divergence of its hosts may provide us some valuable insights. As the host range of *Giardia *extends from amphibians to mammals, we identified orthologous relationships for all proteins from human to mouse, platypus and fish by InParanoid [36]. We constructed a phylogenetic tree based on the VSP homologs (Materia and Methods. Additional file 3: the phylogenetic tree). Based on amino acid similarities, we found that about 60% of VSPs have higher amino acid similarities than the VSP homologs in human and platypus, while 78% of VSPs have higher amino acid similarities between VSP homologs in human and fish.

**Figure 4:**
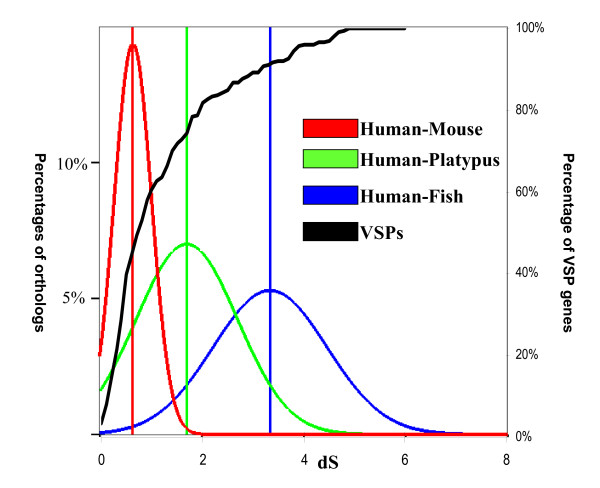
**Comparison of dS for duplicated genes in *G. lamblia *with orthologs among animals**. The red curve represents distribution of dS of orthologs between Human and Mouse; the green curve denotes distribution of dS of orthologs between Human and Platypus; the blue one displays distribution of dS of orthologs between Human and Zebrafish. The black curve is the cumulative distribution of VSP genes in *G. lamblia*.

Many recent duplicated genes underwent positive selection, which would accelerate the evolutionary rate of proteins [37]. dS is presumably considered to be neutral during evolutionary process [37,38]. Thus, we compared the dS values of VSPs with the synonymous substitution rates of orthologous genes from fish to human. As shown in figure 4, about half of the VSPs have smaller dS values than the average dS values of orthologs between human and mouse, and approximately 80% of such duplicated genes have a dS value smaller than the average dS between human and platypus. Although the evolutionary rates in unicellular organisms is more rapid than multi-cellular organisms, for the reason that unicellular organisms usually have shorter generation time, it would be conservative to conclude that VSPs were rapidly expanded in *G. lamblia *after the period in which platypus separated from the ancestor of human and mouse. This time scale is almost consistent with the evolutionary radiation of placental mammals [39-41]. Therefore, it is available to propose a hypothesis that the increment of *Giardia *hosts is the driving force for the rapid expansion of VSPs.

## Discussion

Gene duplication is one of important mechanisms to provide neo-function for adaptive evolution. Systematic surveys of gene duplication in the intestinal parasite *G. lamblia *provided much more information than what we had previously expected. Although many biological machines in *G. lamblia *are considerably compact [8], more than 40% of the genes in *G. lamblia *were identified as duplicated genes in our analysis. This proportion of duplicated genes is similar with those in fly and yeast using the same parameters for BLASTP [42]. Interestingly, we found that a large number of duplicated genes were focused within two large duplicated groups. Further analysis of the two large duplicated groups indicated that there were two rounds of large scale gene duplication events in the evolutionary process of *G. lamblia*. Based on dN values of identified duplication genes, the first round of duplication events happened a long time ago. Due to the extended time of divergence among these duplicated genes, dS and even dN values would be saturated by mutations. Therefore, perhaps the accumulation of ancient duplication genes might result from a saturation of mutations at non-synonymous sites. However, the dS for most of the genes from the second round duplication events have a relatively small dS value (< 1), which could fall short of the saturation of mutations [29] (Additional file 4: the distribution of dS for the ancient and recent duplicated genes). Gene conversion would also result in very high similarities among duplicated genes [37]. Nevertheless, based on current knowledge, the mechanism for gene conversion is still unresolved in *G. lamblia *[43]. Thus, these recent duplication events might be authentic and correspond to the adaptive evolution in *G. lamblia*.

Antigenic variation is an essential mechanism allowing parasitic pathogens to escape from arrest of the host immune system. *G. lamblia *performs this variation by changing the expression of its VSP genes in a variety of hosts at different time points [23,24,44-46]. Our results showed that 74% of the genes in the second round of duplication are VSP genes. Furthermore, in comparison with other parasitic protists, we found that VSP genes expanded independently in *G. lamblia *genome (Additional file 5: gene number in each family in the five studies parasitic protists), even when considering its very close relative *Spironucleus salmonicida *[40]. A probable explanation for the recently rapid expansion of VSP genes in the genome of *G. lamblia *is that the dramatic expansion was driven by the selection of evasion from the host immune systems. Since most of the hosts for *G. lamblia *are animals, we analyzed the possible relationships between the evolution of VSPs and the evolutionary rates for orthologous genes from fish to human. Given the shorter generation time in unicellular organisms compared to the multi-cellular organisms, we inferred that at least VSP genes became expanded in *G. lamblia *after the separation of platypus from the ancestor of placental mammals. At the same time species in mammals also expanded after the divergence from platypus [39-41]. Our results indicate an interesting co-evolution pattern for the parasitic *G. lamblia *in mammals' evolutionary processes. Further, analysis at the genomic level would shed more lights on the understanding of the co-evolution between the parasite and hosts.

## Conclusion

Gene duplication always plays a pivotal role for the adaptive evolution of organisms under changing environments. Although *G. lamblia *is one of the most primitive eukaryotes, the origin of its parasitic lifestyle is not as long as it's surveillance. Global identification of duplicated genes in the genome of *G. lamblia *indicated that gene duplication was essential for the origin and evolution of its parasitic lifestyle. Our results advocated that the recent expansion of VSPs uniquely took place in *G. lamblia*. Comparison of the evolution of VSPs with the divergence of its hosts indicated that the rapid expansion of VSPs is consistent with the increment of its hosts. Therefore we proposed a hypothesis that the increment of *Giardia *hosts is the driving force for the rapid expansion of VSPs.

## Methods

### Sequence data

Protein sequences for all ORF in *Giardia lamblia *[Gla] were downloaded from GiardiaDB database [47]. Perl script was used to filter overlapped ORF which has 80% overlap on the same strand in a contig with another longer ORF. Finally, 5,986 ORFs were used to do analysis. Protein sequences for protists *Cryptosporidium parvum *[Cpa], *Entamoeba histolytica *[Ehi], *Leishmania major *[Lma] and *Plasmodium falciparum *[Pfa] were downloaded from NCBI database [48]. Protein sequences for Human, Mouse, Platypus and Zebrafish were downloaded from Ensembl [49].

### Duplicated genes in *G. lamblia*

In order to detect all possible duplicated genes in *G. lamblia*, all-against-all blast search for 5,986 studied proteins in *G. lamblia *were done by BLASTP program with a loose parameter E-value < 10^-4^. 2,403 proteins which have significant hits with another protein were defined as duplicated genes. The rest (3,583 genes) are single genes. Protein identities between each duplicated pair were used to do cluster analysis by agglomerative hierarchical clustering algorithms (AGNES) in R cluster package. Briefly, a symmetric matrix with 2,403 rows (each one represents a duplicated gene) and 2,403 columns was constructed. And then the amino acid similarities for each gene pair in the row and column were extracted from the BLASTP results. Based on this matrix, we used average method in AGNES to do cluster. Proteins will be clustered closer if they have higher protein similarities. Group I and Group II were defined based on the cluster results. Apart from Group I and II, the rest of the proteins were put together as Group III.

### Evolutionary distance of duplicated genes

In order to estimate evolutionary distance of duplicated genes, we used a reverse-searching method to identify a putative parental gene for each duplicated gene. Briefly, based on BLASTP results, the duplicated pairs with the highest similarity were selected from all of duplicated pairs. For the two copies in each duplicated pair, if the first copy has higher similarities with other genes than the second copy, the first copy would be defined as parental gene for the second copy. After this, the defined daughter gene was removed from all duplicated pairs. Then we iterated this process until we can not find daughter gene at all. Finally, we identified 1,906 duplicated pairs with parental-daughter relationships. The software YN00 in the package PAML was used to estimate the synonymous distance (dS) and non-synonymous distance (dN) for each of the duplicated pairs with parental-daughter relationships[29].

### Functional domain annotation and Gene family identification

Functional domains for all of the duplicated genes have been detected based on the Pfam database. The sequences for duplicated genes were used as queries to search the Pfam_fs database by the hmmpfam program in the HMMER package 2.3.2 [50]. The cut-off for the search was chosen at E-value < 0.1 according to the advice of the author for HMMER. Finally 1,767 genes were annotated as containing the known functional domain, 636 genes do not contain the known functional domain and were annotated as Hypothesis proteins. In order to study gene family expansion in *G. lamblia*, Tribe-MCL was used to do family classification among *G. lamblia *and other four parasitic protists (*Cryptosporidium parvum, Entamoeba histolytica*, *Leishmania major *and *Plasmodium falciparum*).

### Comparative evolution analysis of VSPs

Initially using human genes as queries, we identified all orthologous relationships of genes in human with genes in mouse, platypus and zebrafish. Synonymous substitution rates of one-to-one orthologs were used to estimate divergence among these species. The software YN00 in the package PAML was used to estimate the synonymous distance (dS) for all orthologous pairs [29]. The best hit of VSPs in human, mouse, platypus and zebrafish were identified as VSPs homologs. Then the VSPs gene and its homologs were used to construct the phylogenetic tree. All protein sequences were aligned by MUSCLE [51]. The tree was constructed by Clustalw2 after alignment [52].

## Authors' contributions

JS designed the study. HJ analyzed the data. JS, HJ, RF and JW wrote and revised the manuscript. All authors read and approved the final manuscript.

## Supplementary Material

Additional file 1 **The list of 2,403 duplicated genes**. This table listed all identified duplicated genes in *G. lamblia*. Click here for file

Additional file 2 **List of the ratio of RDG and ADG in each gene family**. This table listed the proportion of RDG and ADG in each identified gene family. Click here for file

Additional file 3 **The phylogenetic tree of VSPs and their homologs**. The amino acid similarities of VSP homologs were listed. The numbers on each branch show the similarities between human and species in the branch. Click here for file

Additional file 4 **The distribution of dS for the ancient and recent duplicated genes**. The dS distribution of all proteins in *G. lamblia *including RDG and ADG were depicted in the figure. Click here for file

Additional file 5 **Distribution of gene families among five studied parasitic protists**. In this table, we presented the distribution of gene family members among five studied parasitic protists. Click here for file
