# Supplementary figures and images for: Gene duplication in the genome of parasitic Giardia lamblia
Source: BMC Evol Biol. 2010 Feb 17;10:49. doi: 10.1186/1471-2148-10-49 (PMC2829556; doi:10.1186/1471-2148-10-49)

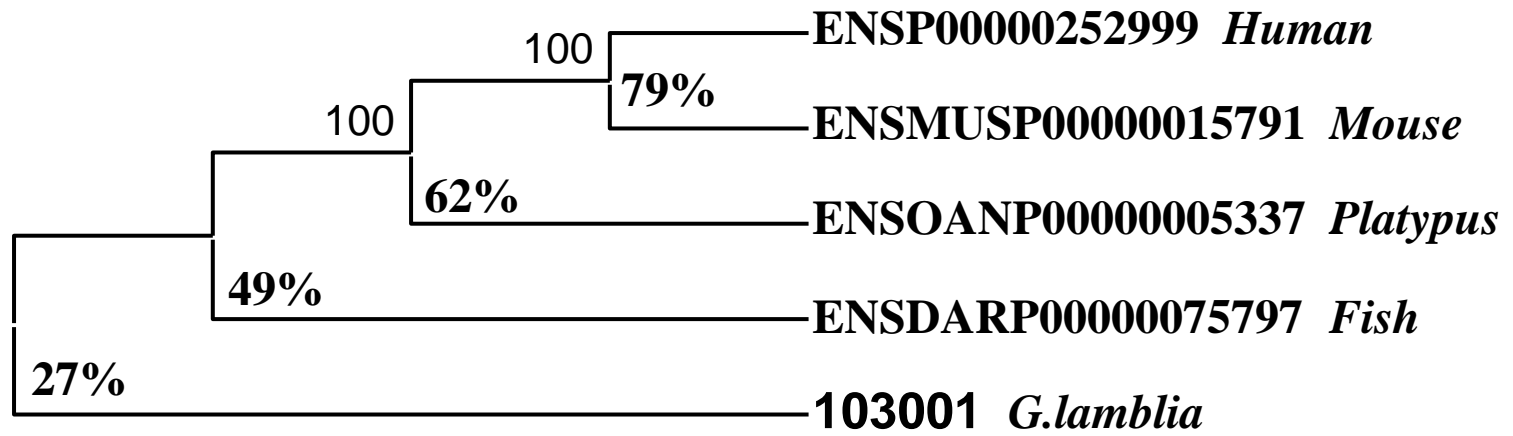

Supplement: Additional file 3 — The phylogenetic tree of VSPs and their homologs. The amino acid similarities of VSP homologs were listed. The numbers on each branch show the similarities between human and species in the branch. [file 1471-2148-10-49-S3.PDF]

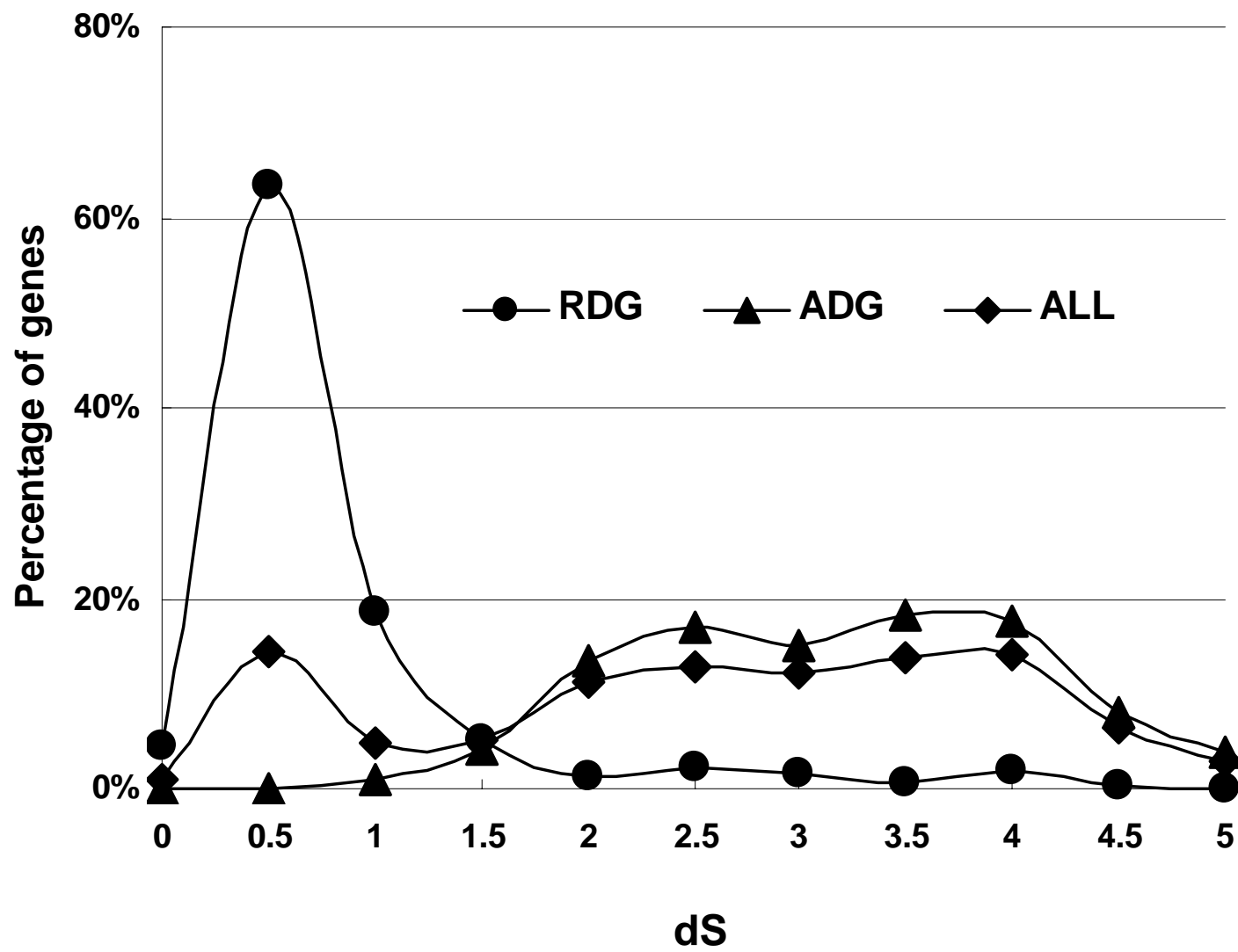

Supplement: Additional file 4 — The distribution of dS for the ancient and recent duplicated genes. The dS distribution of all proteins in G. lamblia including RDG and ADG were depicted in the figure. [file 1471-2148-10-49-S4.PDF]
